# Study on bacterial pathogens through multiplex polymerase chain reaction system and their antimicrobial resistance pattern in goats presumed with fever and/or diarrhea

**DOI:** 10.14202/vetworld.2021.1080-1092

**Published:** 2021-05-06

**Authors:** Pranab Paul, Md. Rayhan Faruque, Md. Kaisar Rahman, Priyanka Das, Mohammed Yousuf Elahi Chowdhury

**Affiliations:** 1 Department of Medicine and Surgery, Chattogram Veterinary and Animal Sciences University, Chattogram, Bangladesh; 2 EcoHealth Alliance, New York, NY, 10018 USA; 3 Faculty of Veterinary Medicine, Chattogram Veterinary and Animal Sciences University, Chattogram, Bangladesh

**Keywords:** antimicrobial resistance, goat, infectious disease, multiplex polymerase chain reaction, prevalence

## Abstract

**Background and Aim:**

Goat is one of the major livestock species, plays an important role in the economy of Bangladesh. However, the outbreak of different infectious diseases in goats causes high mortality and economic losses due to lack of proper diagnosis and treatment. Conventional culture-based methods for detecting specific pathogens as confirmatory diagnosis are laborious as well as time-consuming in comparison to multiplex polymerase chain reaction (mPCR), by which multiple pathogens can be detected at a time. The present study was aimed to perform faster molecular identification of bacterial pathogens from goats presumed with fever and/or diarrhea and their antimicrobial resistance (AMR) pattern.

**Materials and Methods:**

A total of 200 blood samples were collected from goats at S. A. Quaderi Teaching Veterinary Hospital (SAQTVH) in Chattogram Veterinary and Animal Sciences University for the period of July 2017-April 2018. DNA was extracted and subsequently, mPCR assay was performed for the screening of several bacterial pathogens (*Salmonella* spp., *Listeria monocytogenes*, *Bacillus cereus*, *Yersinia enterocolitica*, *Campylobacter jejuni*, *Campylobacter coli*, *Clostridium perfringens*, *Vibrio cholerae*, and *Staphylococcus aureus*). An antimicrobial susceptibility test against ten antimicrobials for positive samples of each organism was conducted using the Kirby–Bauer Disk-Diffusion Method on selective media.

**Results:**

*S. aureus*, *C. perfringens*, *L. monocytogenes*, and *Salmonella* spp. were detected from collected samples and their overall prevalence was 11.5%, 3.5%, 1%, and 20.5%, respectively. The most common clinical signs were mild fever, nasal discharge, dyspnea, and coughing (39.1%) for *S. aureus*, diarrhea, convulsion, abdominal pain, and incoordination (57.1%) for *C. perfringens*, fever, protrusion of tongue, and incoordination (100%) for *L. monocytogenes*, and fever, anorexia, dehydration with mucous feces (36.6%) for *Salmonella* spp. infection in goats. AntimGentamicinicrobial diagram of *S. aureus* showed resistance against Cefotaxime (74%), Cefixime (65%), and Tetracycline (65%); highly sensitive against Amoxicillin (48%), Ciprofloxacin (44%), and Gentamicin (44%). On the other hand, *C. perfringens* showed highly resistant against Ampicillin (71%), Gentamicin (71%), sensitive against Penicillin (57%), and Cefotaxime (57%). *L. monocytogenes* were found to be sensitive to Penicillin (100%) and Cefixime (100%) and *Salmonella* spp. showed resistance to Ampicillin (78%) and Amoxicillin (59%) but sensitive to Ciprofloxacin (54%).

**Conclusion:**

This study identified pathogens with their specific clinical signs in goats presumed fever and/or diarrhea through mPCR with their AMR pattern in SAQTVH, Chattogram. Potential risk factors, measuring the strength of association of disease caused by these particular pathogens, were also determined. mPCR may use as an effective tool for rapid detection of pathogens in animal.

## Introduction

Small ruminants, especially goats are the most important contributor in rural economy and nutrition using as a tool for poverty alleviation in Bangladesh. As far as known, goats are probably the first domesticated animal on Earth [1]. In this subcontinent, about 300 breeds and varieties of goats are found [2]. In Bangladesh, more than 90% are Black Bengal and the remaining are Jamunapari and their crosses [3]. Goats are ideally suited for poor people who have no ability to buy and rear large ruminants. As a result, goat husbandry is becoming an attractive activity among poor women [4] under the traditional scavenging system but still contributing to the rural economy through income generation, women empowerment, and hence rightly treated as an effective tool for the reduction of poverty. Goats are performing a variety of functions and ranks second in position in terms of meat, milk, and skin production, representing about 28, 23, and 28%, respectively, of total livestock in Bangladesh [5].

However, different infectious diseases are the most important constraint of rearing goats in Bangladesh. Among them, viral diseases such as Peste Des Petits Ruminants (PPR), goat pox, contagious ecthyma, and viral pneumonia, bacterial diseases such as enterotoxaemia, tetanus, brucellosis, mastitis, and metritis, mycotic diseases such as ringworm infection, and rickettsial disease such as conjunctivitis are commonly found causing morbidity as well as mortality of goats. Gastro-intestinal nematodiasis, fascioliasis, and tapeworm are also common, causing severe depression in the growth and reproduction rate [6]. High density of animals and seasonal variation particularly the long monsoon period are two main reasons responsible for the occurrence of diseases. Moreover, the climatic condition is also conducive to grow the pathogens and causing diseases in this area. However, the total disease complex is not yet clear, due to the general lack of standardized diagnostic and disease recording services in the department of livestock services.

For the diagnosis of diseases, characteristics clinical signs, physical examination findings, and some conventional diagnostic methods are used till now. For the confirmatory diagnosis of bacterial infection, detection of specific bacterial pathogens is done by conventional method of culturing microorganisms on agar plates, followed by standard biochemical identifications [7]. These methods are usually inexpensive and simple but time-consuming as they depend on the ability of the microorganisms to grow in different culture media such as pre-enrichment media, selective enrichment media, and selective plating media [8]. It requires 2-3 days for preliminary identification and more than a week for the confirmation of species of the pathogens [9]. Moreover, these methods are laborious as they require too many procedures to be accomplished. Furthermore, false-negative results may found due to viable but non-culturable pathogens [10]. The failure of pathogen detection would increase the transmission risk of pathogens and treatment failure. Most importantly, delays in specific diagnosis followed by choosing of wrong antimicrobials may cause poor health care quality as well as increase antimicrobial resistance (AMR). Therefore, different rapid diagnostic methods with high sensitivity and specificity have been developed to improve the detection and identification of pathogens. Rapid methods are also more time-efficient, labor-saving, and able to reduce human errors [11]. One of the most commonly used molecular-based methods for the detection of bacterial pathogens is multiplex polymerase chain reaction (mPCR). It offers more rapid detection of multiple gene targets though the basic principle is similar to the conventional PCR [10]. It can also reduce the use of antimicrobial agents in addition to allowing a faster switch to the most optimum treatment, thus reducing both side effects and costs [12,13]. However, using these rapid diagnostics kits are not yet established to diagnose the non-specific diseases in livestock in Bangladesh. Moreover, the practitioners are not aware enough of using specific antimicrobial to combat such kind of infectious diseases at the field level.

Therefore, the current study was aimed to confirm the specific causal agents causing non-specific clinical signs through mPCR and to assess the effectiveness of it in clinical diagnosis. In addition, the prevalence of these diseases along with their probable risk factors and the status of antimicrobial using against those infectious diseases in goats were determined.

## Materials and Methods

### Ethical approval and Informed consent

Ethical approval was not necessary for this study; however, samples were collected as per standard sample collection procedure and consent was taken from the animal owners with their signature using a prescribed consent form.

### Study period and location

The study was conducted from July 2017 to April 2018. The study was conducted on goats brought to S. A. Quaderi Teaching Veterinary Hospital (SAQTVH) of Chattogram Veterinary and Animal Sciences University (CVASU).

### Study design and sampling

In Chattogram Metropolitan area, goats are usually reared in backyard household or semi-intensive farming management systems and no significant sanitary measures are usually practiced. However, the two common feeding practices are found to be adopted by the household farmers that are grazing without any supplement and grazing with supplement. Two hundred (n=200) blood samples were collected from the goats having fever and/or diarrhea. The blood samples from goats only having diarrhea were collected after ruling out the dietary and parasitic causes. Samples were collected from the jugular vein. Collected samples were placed in a vacutainer (5 mL) containing ethylenediaminetetraacetic acid (EDTA), and transported with 4°C cool box to the Poultry Research and Training Centre (PRTC), CVASU for further analysis. All the molecular investigations of this study were conducted in PRTC, CVASU.

### Questionnaire used for sample collection

A pre-designed questionnaire was used to collect relevant information such as species, age, and sex, diarrheic or not, and antibiotic used or not from the sampled goats. An attempt was made to enlist the antimicrobials used for that specific case. Recovery after using the drugs against infection was noted during the study.

### DNA extraction from blood samples

DNA was extracted from collected blood samples using DNA extraction kit (K-3000, GeNet Bio, Korea) according to the method described elsewhere [14]. Briefly, Proteinase K solution (20 mL, 20 mg/mL) was added to a 1.5 mL microcentrifuge tube. Then, whole blood sample (200 mL) was transferred to the microcentrifuge tube. Buffer GB (200 mL) was added to the sample and mixed by vortexing for 15 s and incubated at 56°C for 10 min. After that, absolute ethanol (200 mL) was added and again mixed by vortexing for 15 s. The tube was then briefly centrifuged to get the drops clinging under the lid. Afterward, the lysate was carefully transferred into the upper reservoir of the spin column (fit in a 2 mL tube) without wetting the rim and centrifuged at 10,000 rpm for 1 min. The spin column was transferred to a new 2 mL collection tube and washed 2 times with buffer GW1 (500 mL), followed by centrifugation at 10,000 rpm for 1 min. Furthermore, for complete removal of ethanol, it was centrifuged once again at 12,000 rpm for 2 min and ensures that there was no droplet clinging to the bottom of the collection tube. For elution, column was transferred to a new 1.5 mL tube, then buffer GE (200 mL) was added and waited for 1 min at room temperature. Finally, DNA was eluted by centrifugation at 10,000 rpm for 1 min.

### mPCR reactions

mPCR was conducted according to the instruction given by the manufacturing company (EB-1000, GeNet Bio, Korea). The primer sequences used for the mPCR are shown in Table-1. The mPCR reactions were conducted with a final volume of 20 μL. Proportions of different reagents used for mPCR of different genes are given in Table-2. The PCR reaction was run in a thermocycler (Applied Biosystem, 2720 thermal cycler, Singapore) following the cycling conditions mentioned in Table-3. The mPCR protocol followed by the agarose gel electrophoresis technique was adopted for the detection of bacterial genes from whole blood as described by Sambrook and Russell [15].

**Table-1:** Primer sequence of bacterial toxin genes.

Bacteria	Toxins	Primer sequence	Size
Lambda DNA	lambda-F	CGCGAATATGCCGGTTATCA	1000bp
	lambda-R	CACGGAGTAGCCGTTATCCGT	
*Salmonella* spp.	invA-F	TCATATTACGCACGGAAACACGTTC	100bp
	invA-R	CCTGATTTACTTAAAGAAGTGCTCAG	
*Lysteria monocytogenes*	prfA-F	GGAGTTTCTTTACCATACACATAGGTC	150bp
	prfA-R	TCTTACGCACTTTTTCTATGTTTTCCAAA	
*Bacillus cereus*	hblC-F	CTCTCGCAACACCAATCGTTCA	200bp
	hblC-R	CCATTCCTTCATATCTTGTTTGATTAG	
	bceT-F	TTCAGTTCAAAGAAGCATGGACGAAAG	
	bceT-R	ATGCTGACGAGCTACATCCATAATGACT	
	nheA-F	ACAGGGTTATTGGTTACAGCAGTATC	
	nheA-R	TCTGGCTGTTGCAAAATAAYTAATCC	
	entFM-F	TGTTCGTTCAGGTGCTGGTACAGG	
	entFM-R	ACTGTGTAAGTACCWGTTCCTTGTTGAA	
	cytK-F	AGGGATTGGGTAGTTATCAATAGG	
	cytK-R	TCGGGCAAAATGCAAAAACACATACG	
	CER-F	GGGACCAAGAAACGAAAAAGAAGCA	
	CER-R	AGTTCAGCAATCGTTTGATACTGAAAG	
*Yersinia enterocolitica*	inv (Y)-F	GGCAAATCAGGAAGTAAAACACTGG	250bp
	inv (Y)-R	TGTCATAGAAAGTGTTAAAGCCATAC	
*Campylobacter jejuni*	hipO-F	TCTGGAGCACTTCCATGACCACC	300bp
	hipO-R	TTGCGGTCATGATGGACATACTAC	
*Campylobacter coli*	glyA-F	TCAAGGCGTTTATGCTGCACTTTTAA	
	glyA-R	GCAATGTCTGCAAAAAGATAAGCTCCAAC	
*Clostridium perfringens*	cpe-F	TGGATTTGGAATAACTATAGGAGAAC	400bp
	cpe-R	AGTCCAAGGGTATGAGTTAGAAGAACG	
	cpb2-F	AGCAATAAGTCCAATGAAAGCAAGTGC	
	cpb2-R	ACAAACTTGAGTTCTAAATGATGGTGT	
*Vibrio cholera*	hly-F	AGCAGAGATGCAAGCCCAATTCAG	500bp
	hly-R	TGGCTCCAAACTGACGATAACCGAG	
*Vibrio vulnificus*	vvha-F	GGGTATTTGATAAGACGAAGTTCAA	
	vvha-R	CTAAGTTCGCACCACACTGTTCG	
*Vibrio parahaemolyticus*	tlh-F	TCGCACCAGCTACTCGAAAGATG	
	tlh-R	CAACCCCTGTTAGCGCGATGTATT	
*Staphylococcus aureus*	nuc-F	GTGCTGGCATATGTATGGCAAT	658bp
	nuc-R	CTGAATCAGCGTTGTCTTCGC	

**Table-2:** Contents of each reaction mixture of Multiplex Polymerase Chain Reaction assay.

Components	Volume
2 Multi HS Prime Taq Premix	10 μL
Primer Mixture	5 μL
Template DNA	5 μL
Total	20 μL

**Table-3:** Cycling conditions used during multiplex polymerase chain reaction for detection of bacterial genes.

Step	Temperature	Time	Cycle
UDG reaction	50°C	3 min	1
Pre-denaturation	95°C	10 min	1
Denaturation	95°C	30 s	35
Annealing	60°C	20 s	
Extension	72°C	1 min	
Final extension	72°C	5 min	1
Store	4°C	∞	-

### Visualization of mPCR products by agar gel electrophoresis

Agarose gel (1.5 %, W/V) was used to visualize the PCR product. Agarose powder (0.75 g) and 50 mL of 1× TAE buffer were mixed thoroughly in a conical flask and boiled in a microwave oven until dissolved. Then, the mixture was cooled to 50°C using a rocker and 0.05 μL/mL of ethidium bromide was added to the agarose solution. The agarose solution was poured into the gel casting tray which was previously assembled and sealed. Gel was kept at room temperature (25ºC) for solidification and transferred into an electrophoresis tank filled with 1× TAE buffer for further use. An amount of 7 μL of PCR product and 1 kb control DNA marker was loaded into a gel hole to compare the size of the amplicons. Finally, electrophoresis was run at 110 v and 80 mA for 30 min, allowed to go down to the target level and then viewed in ultraviolet trans-illuminator in the dark chamber for image viewing and documentation system.

### AMR profile testing of bacteria

The AMR pattern of the isolated organism was tested against the ten most commonly used antimicrobials in SAQTVH. Positive samples were sub-cultured on blood agar and incubated at 37°C for 24 h to obtain pure growth. Using a sterile inoculating loop, three or four individual colonies from the blood agar were transferred into a tube containing 3 mL of sterile phosphate-buffered saline (PBS) solution (PBS, 0.85% w/v NaCl solution). Emulsification of the inoculum was done to avoid clumping of the cells inside the test tube using a vortex machine. Then, the bacterial suspension was adjusted to the turbidity of 0.5 McFarland standards (equivalent to growth of 1-2×10^8^CFU/mL). Within 15 min of preparing the inoculums, a pre-sterile cotton swab was dipped into the inoculums and rotated against the side of the tube with firm pressure to remove excess fluid. The swab was streaked over the entire dry surface of Mueller Hinton agar by rotating 3 times at approximately 60°F. After 15 min of inoculation, disks were placed on the agar surface using sterile forceps. Agar plates were incubated at 37°C for 18 h after dispensing all the discs on it. The size of zone of inhibition (in mm) around a disk including the diameter of disk was measured using a ruler and the result was interpreted according to CLSI, 2011 [16]. The panel of antibiotics used for different bacterial species along with the size of zone of inhibition of them to be considered as resistant (R), intermediately sensitive (I), and sensitive (S) against the tested isolates are shown in Table-4.

**Table-4:** Panel of antibiotics, their concentration, and zone diameter interpretative standards for different bacteria (CLIS, 2011).

Name of bacteria	Name of Antimicrobial agent (mg)	Disk content (mg)	Interpretation of zone diameters (mm)		

			R≤	I	S≥
*Salmonella* spp.	Penicillin	6	-	-	-
	Ampicillin	10	14	-	15
	Amoxicillin	25	14	-	15
	Cefotaxime	30	19	-	20
	Gentamicin	10	16	17-19	20
	Ciprofloxacin	5	16	17-19	20
	Tetracycline	3	19	20-23	24
	Sulfamethoxazole	300	13	14-16	17
*Listeria monocytogenes*	Penicillin	6	28	20-27	19
	Ampicillin	10	20	-	19
	Amoxicillin	25	-	-	-
	Cefotaxime	30	-	-	-
	Gentamicin	10	-	-	-
	Ciprofloxacin	5	-	-	-
	Tetracycline	3	19	15-18	14
	Sulfamethoxazole	300	16	11-15	10
*Clostridium perfringens*	Penicillin	6	12.5	1.6-6.2	0.8
	Ampicillin	10	-	-	-
	Amoxicillin	25	-	-	-
	Cefotaxime	30	-	-	-
	Gentamicin	10	-	-	-
	Ciprofloxacin	5	-	-	-
	Tetracycline	3	12.5	3.1-6.2	1 0.6
	Sulfamethoxazole	300	-	-	-
*Staphylococcus aureus*	Penicillin	6	24	-	25
	Ampicillin	10	25	-	26
	Amoxicillin	25	-	-	-
	Cefotaxime	30	21	-	22
	Gentamicin	10	-	-	-
	Ciprofloxacin	5	13	-	14
	Tetracycline	3	19	-	20
	Sulfamethoxazole	300	19	-	20

R=Resistant, I=Intermediate S=Sensitive, (-)=No established value found

### Statistical analysis

The data were entered into Microsoft Office Excel 2013 and then exported to STATA-13 (StataCorp 4905, Lakeway Drive, College Station, Texas 77845, USA) for epidemiological analysis.

#### Descriptive analysis

The distribution of goats was presented according to the locations and quantities of the group, population size, sample size, age, and sex variables. The prevalence of different microorganisms was calculated using positive samples divided by the total number of samples tested and the results were expressed as a percentage with 95% confidence interval (CI). Similarly, the prevalence of microorganisms according to the season, sexes, and age was also calculated. Antimicrobial susceptibility testing (AST) was done and the percentage of susceptibility was calculated as resistance, intermediate, and sensitive. Percentages of different antimicrobials were presented as a chart.

#### Risk factor analysis

Based on data collection, goat samples were grouped according to the seasons (summer and winter), breed (Black-Bengal, Jamunapari, and Cross), source (family and farm), age (adult, sub-adult, juvenile and young), sex (male and female), and body condition score (BCS) and were classified following the Vieira *et al*. [17], (poor, fair, and good) and PPR vaccination (yes and no). A Chi-square test was done to identify significant risk factors.

#### Logistic regression model

For *Staphylococcus* *aureus* and *Clostridium perfringens*, variables – Breed, sex, and age (p<0.3) were forwarded to the logistic regression model after the Chi-square test. In the case of *Salmonella* spp., sex and age were dropped and source was added. Logistic regression was omitted in case due to the low prevalence. After adjusting the factor with each other *S. aureus* (breed, sex, age, and BCS), *C. perfringens* (breed, sex, and age), and *Salmonella* spp. (breed, source, BCS, and vaccination), were found to be a significant risk factor. Confounder was checked by observing the variation in the coefficient. If the variation was >10%, then the factor was considered as a confounder. The validity of the model was checked. The model was valid by the receiver operating curve and goodness of fit test (lfit) [18]. The results were expressed as OR, 95% CI, and p-value.

## Results

### Confirmation and prevalence of pathogens by mPCR

**Figure-1:**
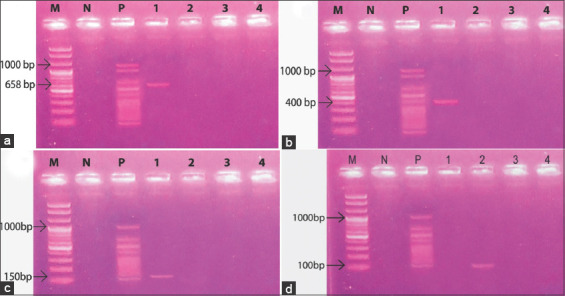
Result of multiplex polymerase chain reaction assay for (a) nuc gene of Staphylococcus aureus identified from the samples; Lane M: 1 Kb DNA marker; Lane N: Negative control; Lane P: Control DNA; Lane 1: Staphylococcus aureus gene-sized (658 bp) amplicon, (b) cpe and cpb2 gene of *Clostridium perfringens* identify from the samples; Lane M: 1 Kb DNA marker; Lane N: Negative control; Lane P: Control DNA; Lane 1: *C. perfringens* gene-sized (400 bp) amplicon, (c) prfA gene of *Listeria monocytogenes* identify from the samples; Lane M: 1 Kb DNA marker; Lane N: Negative control; Lane P: Control DNA; Lane 1: *L. monocytogenes* gene-sized (150 bp) amplicon, (d) invA gene of Salmonella spp. identify from the samples; Lane M: 1 Kb DNA marker; Lane N: Negative control; Lane P: Control DNA; Lane 2: Salmonella spp. gene-sized (100 bp) amplicon.

A total of 200 samples were collected from goats having fever and/or diarrhea brought to SAQTVH, CVASU from different parts of Chattogram metropolitan areas and pathogens were identified through mPCR followed by electrophoresis. *S*. *aureus*, *C. perfringenes*, *Listeria monocytogenes*, and *Salmonella* spp. were confirmed by observing their band size as 658 bp (Figure-1a), 400 bp (Figure-1b), 150 bp (Figure-1c), and 100 bp (Figure-1d), respectively. Among them, 23 (11.5%; 95% CI 7.4%-16.7%) were *S*. *aureus*, 07 (3.5%; 95% CI 1.4%-7.1%) were *C. perfringenes*, 2 (1%; 95% CI 0.1%-3.6%) were *L. monocytogenes*, and 41 (20.5%; 95% CI 15.1%-26.8%) were confirmed as *Salmonella* spp. (Table-5).

**Table-5:** Prevalence of microorganisms confirmed by multiplex polymerase chain reaction.

Name of the microorganisms	Positive (n)	Prevalence	95% CI
*Staphylococcus aureus*	23	11.5%	7.4-16.7
*Vibrio cholera*	0	0	-
*Clostridium perfringens*	7	3.5%	1.4-7.1
*Campylobacter jejuni*	0	0	-
*Yersinia enterocolitica*	0	0	-
*Bacillus cereus*	0	0	-
*Listeria monocytogenes*	2	1%	0.1-3.6
*Salmonella* spp.	41	20.5%	15.1-26.8

### Frequency of different clinical signs and associated factors influencing the *S. aureus* infection in goats

Major clinical signs were recorded while collecting samples from goats having diarrhea and/or fever. The *S. aureus* infected goats were showing variable clinical signs and symptoms. Percentages of major clinical signs were calculated and found that mild fever, nasal discharge, dyspnea, and coughing are the most common clinical signs of *S. aureus* infection (39.1%). Goats with a history of parturition having fever, lethargy, dehydration, and loss of appetite (34.8%), followed by mild fever, dry nose, and wheezing (17.4%) and fever, sneezing, foaming at the mouth (8.7%), were also recorded in *S. aureus* infection in goats (Table-6). From these findings, we can come into the conclusion that, goat infected with *S. aureus* may show common clinical signs of fever and respiratory distress such as coughing, dyspnea, and nasal discharge.

**Table-6:** Frequency distribution of symptoms due to infection of *Staphylococcus aureus* in goats.

Clinical signs and symptoms	N	%
Mild fever, nasal discharge, dyspnea, and coughing	09	39.1
Fever, lethargy, dehydration, loss of appetite, and parturition history	08	34.8
Mild fever, dry nose, and wheezing	04	17.4
Fever, sneezing, and foaming at the mouth	02	8.7

In this study, among *S. aureus* positive goats, the prevalence rate was found to be significantly (p≤0.01) higher in female (16.8%; 95% CI: 10.6-24.7) than the male (3.7%; 95% CI: 0.7-10.4). Adult goats (33.3%; 95% CI: 18.6-50.9) were more prevalent than that of young (9.8%; 95% CI: 4.1-19.3) and juvenile (4.3%; 95% CI: 1.2-10.6) which was also statistically significant (p≤0.01). In the case of breed, the highest prevalence of *S. aureus* was found in Jamnapari (14.3%; 95%, CI: 8.8-21.4) in comparison to Crossbreed (7.1%; 95% CI: 1.5-19.5) and Black Bengal (4%; 95% CI: 0.1-20.3) breeds of goats which was not statistically significant. Surprisingly, goats with good BCS was more prevalent (18.5%; 95% CI: 9.3-31.4) to the *S. aureus* infection than fair (9.8%; 95% CI: 5.5-21.1) and poor (4.3%; 95% CI: 1.6-14.2) BCS goats (Table-7). In multivariable logistic regression analysis, it is revealed that the prevalence of *S. aureus* was 3.6 and 2.4 times higher in good and fair BCS goats, respectively than the goats having poor BCS. In the case of sex, females were found to be 2.6 times higher in prevalence than males (Table-7).

**Table-7:** Factors influencing the infection of *Staphylococcus aureus* in goats.

Variables	Categories	*Staphylococcus aureus*			Multiple logistic regression		
	
		n (%)	95% CI	p (χ^2^-test)	OR	95% CI	p-value
Season	Summer (80)	11 (13.7%)	7.1-23.3	0.42			
	Winter (120)	12 (10%)	5.3-16.8				
Breed	Black Bengal (25)	1 (4%)	0.1-20.3	0.02	1		
	Jamnapari (133)	19 (14.3%)	8.8-21.4		1.6	0.2-14.2	0.67
	Cross (42)	3 (7.1%)	1.5-19.5		1.5	0.1-18.1	0.71
Sex	Male (81)	3 (3.7%)	0.7-10.4	**<0.01**	1		
	Female (119)	20 (16.8%)	10.6-24.7		2.6	0.6-10.9	0.20
Source	Family (146)	15 (10.3%)	5.8-16.4	0.37			
	Farm (54)	8 (14.8%)	6.6-27.1				
Age	Juvenile (0 days-1 year) (93)	4 (4.3%)	1.2-10.6	**<0.01**	1		
	Young (1 year-2 years) (71)	7 (9.8%)	4.1-19.3		1.8	0.5-6.9	0.39
	Adult (>2 years) (36)	12 (33.3%)	18.6-50.9		6	1.5-24.5	**0.01**
Body condition score	Poor -1 (69)	4 (5.8%)	1.6-14.2	0.09	1		
	Fair-2 (77)	9 (11.7%)	5.5-21.1		2.4	0.6-8.6	0.18
	Good-3 (54)	10 (18.5%)	9.3-31.4		3.6	0.9-13.2	**0.05**
Vaccination	Yes (14)	1 (7.1%)	0.2-33.8	0.59			
	No (186)	22 (11.8%)	7.6-17.4				

### AMR pattern of *S. aureus* in goats

To observe the AMR pattern, cultural sensitivity test was performed against ten different antimicrobials. *S. aureus* showed the highest resistance against Cefotaxime (74%), followed by Cefixime (65%), Tetracycline (65%), and Penicillin (61%) and the lowest resistance against Gentamicin (13%), Amoxicillin (13%), and Ciprofloxacin (18%) in goats.

**Figure-2:**
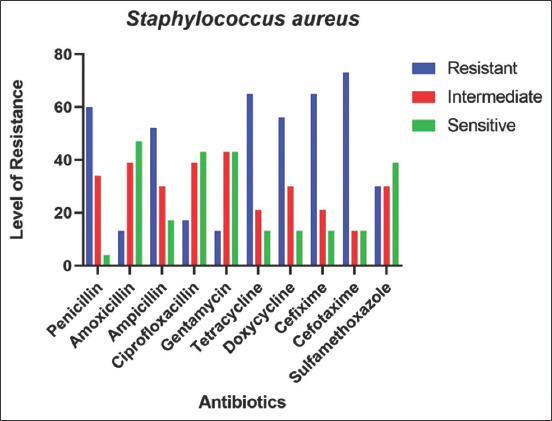
Antimicrobial resistance pattern of *Staphylococcus aureus*.

In the case of the sensitivity of antimicrobials, Amoxicillin (48%), Ciprofloxacin (44%), and Gentamicin (44%) were showing the highest sensitivity, whereas Cefixime (13%), Cefotaxime (13%), Doxycycline (13%), and Tetracycline (13%) showed the lowest sensitivity among all drugs against *S. aureus* infection in goats (Figure-2).

### Frequency of different clinical signs and associated factors influencing the *C. perfringens* infection in goats

Diarrhea, convulsion, abdominal pain, and incoordination (57.1%) were recorded as the most common clinical signs of *C. perfringens* infection in goats. Other important signs presented by *C. perfringens* infected goats were fever, diarrhea, and convulsion (28.6%) and anorexia, diarrhea with blood and dehydration (14.3%) Table-8. From these findings, we can say that goat infected with *C. perfringens* may show common clinical signs of fever, diarrhea, and convulsion. Incoordination and abdominal pain may also be found.

**Table-8:** Frequency distribution of symptoms due to infection of *Clostridium perfringens* in goats.

Clinical signs and symptoms	N	%
Diarrhea, convulsion, abdominal pain, and incoordination	04	57.1
Fever, diarrhea, and convulsion	02	28.6
Anorexia, diarrhea with blood, and dehydration	01	17.1

In risk factor analysis, no significant differences were observed between summer and winter seasons in the prevalence of Clostridial infection in goats*.*However, the prevalence in winter is somewhat higher (4.2%; 95% CI: 1.4-9.5) than summer (2.5%; 95% CI: 0.3-8.7). Within three breeds, the prevalence of *C. perfringens* was significantly higher in the crossbreeds (11.9%, 95% CI: 3.9-25.6, p<0.01) than that of Jamunapari (1.5%; 95% CI: 0.2-5.3) and Black Bengal (0%). Male was most prevalent in *C. perfringens* (7.4%, 95% CI: 2.8-15.4, p=0.01) infection in comparison with females (0.8%; 95% CI: 0.1-4.6). There was no significant difference found in different ages and BCS but a higher percentage of positive was found in juvenile (5.4%; 95% CI: 1.8-12.1) than other age groups and goats with poor BCS showed higher prevalence (4.3%; 95% CI: 0.9-12.2) of *C. perfringens* than fair (2.6%; 95% CI: 0.3-9.1) and good (3.7%; 95% CI: 0.5-12.7) BCS goats (Table-9). In multivariable logistic regression analysis of significant factors, it is revealed that the prevalence of *C. perfringens* infection was 4.9 times higher in crossbreeds than Jamunapari and Black Bengal. In the case of sex, male goats were found to be 5.6 times higher at risk of *C. perfringens* infection than the females (Table-9).

**Table-9:** Factors influencing the infection of *Clostridium perfringens* in goats.

Variables	Categories	*Clostridium* *perfringens*			Multiple logistic regression		
	
		n (%)	95% CI	p (χ^2^-test)	OR	95% CI	p-value
Season	Summer (80)	2 (2.5)	0.3-8.7	0.53			
	Winter (120)	5 (4.2)	**1.4-9.5**				
Breed	Black Bengal (25)	0	0	**<0.01**			
	Jamunapari (133)	2 (1.5%)	0.2-5.3		1		
	Cross (42)	5 (11.9%)	3.9-25.6		4.9	0.8-28.7	0.08
Sex	Female (119)	1 (0.8%)	0.1-4.6	**0.01**	1		
	Male (81)	6 (7.4%)	2.8-15.4		5.6	0.6-54.1	0.13
Source	Family (146)	5 (3.4%)	1.1-7.8	0.92			
	Farm (54)	2 (3.7%)	0.5-12.7				
Age	Juvenile (0 days-1 year) (93)	5 (5.4%)	1.8-12.1	**0.30**			
	Young (1 year-2 years) (71)	2 (2.8%)	0.3-9.8				
	Adult (> 2 years) (36)	0	0				
Body condition score	Poor-1 (69)	3 (4.3%)	0.9-12.2	0.84			
	Fair- 2 (77)	2 (2.6%)	0.3-9.1				
	Good-3 (54)	2 (3.7%)	0.5-12.7				
Vaccination	Yes (14)	0	0	0.46			
	No (186)	7 (3.7%)	1.5-7.6				

### AMR pattern of *C. perfringens* in goats

**Figure-3:**
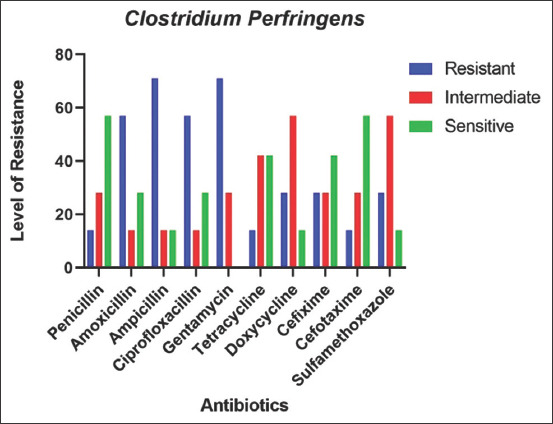
Antimicrobial resistance pattern of *Clostridium perfringens*.

Like *S. aureus*, AMR pattern was also investigated for *C. perfringens* and found that, *C. perfringens* was highly resistant against Ampicillin (71%) and Gentamicin (71%), followed by Amoxicillin (57.1%) and Ciprofloxacin (57.1%) and the lowest resistance against Penicillin (14%), Tetracycline (14.3%), and Cefotaxime (14%) in goats. However, Penicillin (57%) and Cefotaxime (57%) followed by Tetracycline (43%) and Cefixime (43%) were found to be highly sensitive among all the tested antibiotics against *C. perfringens* and Gentamicin (0%) found the least sensitive among all antibiotics tested against this organism (Figure-3).

### Frequency of different clinical signs and associated factors influencing the *L. monocytogenes* infection in goats

In the present study, only two cases of *L. monocytogenes* were identified out of 200 patients. In both cases, fever, protrusion of tongue, and incoordination were recorded as common clinical signs (Table-10). Association between *L. monocytogenes* and selected variables was measured to see the relation of listeria infection with different risk factors. Due to the low prevalence, multiple logistic regressions were not possible to calculate. The prevalence of listeria infection was recorded only in winter (1.7%; 95% CI: 0.2-5.9); none of cases were recorded in summer (0%). Similarly, male (2.5%; 95% CI: 0.3-8.6) Jamunapari (1.5%; 95% CI; 0.1-5.3), from farm sources (3.8; 95% CI: 0.4-13.2%), both from Juvenile (1.1%; 95% CI: 0.03-5.8) and young (1.4%; 95% CI: 0.04-7.6) age groups with poor (2.9%; 95% CI: 0.3-10.1) BCS were found to be prevalent for the infection of *L. monocytogenes* in this study (Table-11).

**Table-10:** Frequency distribution of symptoms due to infection of *Listeria monocytogenes* in goats.

Clinical signs and symptoms	N	%
Fever, protrusion of tongue and incoordination	02	100

**Table-11:** Factors influencing the infection of *Listeria* *monocytogenes* in goats of Chittagong.

Variables	Categories	*Listeria monocytogenes*		

		n (%)	95% CI	p-value (c^2^-test)
Season	Winter (120)	2 (1.7%)	0.2-5.9	0.24
	Summer (80)	0	-	
Breed	Jamunapari (133)	2 (1.5%)	0.1-5.3	0.6
	Black Bengal (25)	0		
	Cross (42)	0		
Sex	Female (119)	0		0.08
	Male (81)	2 (2.5%)	0.3-8.6	
Source	Farm (54)	2 (3.8%)	0.4-13.2	**0.02**
	Family (146)	0		
Age	Juvenile (0 days-1 year) (93)	1 (1.1%)	0.03-5.8	0.78
	Young (1 year-2 years) (71)	1 (1.4%)	0.04-7.6	
	Adults (>2 years) (36)	0		
Body condition score	Poor-1 (69)	2 (2.9%)	0.3-10.1	0.15
	Fair-2 (77)	0		
	Good-3 (54)	0		
Vaccination	Yes (14)			0.69
	No (186)	2 (1.1%)	0.1-3.8	

### AMR pattern of *L. monocytogenes* in goats

**Figure-4:**
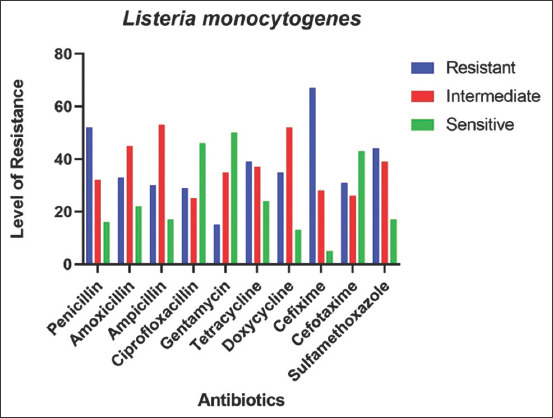
Antimicrobial resistance pattern of *Listeria monocytogenes*.

AMR pattern of *L. monocytogenes* showed higher resistance against Amoxicillin and Ampicillin (100%), followed by Doxycycline, Cefotaxime, and Sulfamethoxazole (50%). However, Penicillin and Cefixime (100%) showed highly sensitivity against *L. monocytogenes* in goats (Figure-4).

### Frequency of different clinical signs and associated factors influencing the *Salmonella* spp. infection in goats

In the case of salmonellosis in goats, fever, anorexia, dehydration, and mucus in diarrheic feces (36.6%) were the most prevalent clinical signs. Profuse watery foul-smelling diarrhea, anorexia with dehydration (29.3%), and high fever, lethargy with yellow to greenish-brown diarrhea (17.1%) were also recorded in salmonella infected goats. From these studies, we can say that fever and diarrhea are common in salmonella infected goat. Diarrhea could be different in color with foul-smelling; blood may or may not be present (Table-12).

**Table-12:** Frequency distribution of symptoms due to infection of *Salmonella* spp. in goats.

Clinical signs and symptoms	N	%
Fever, anorexia, dehydration, and mucus in stool	15	36.6
Profuse, watery foul-smelling diarrhea, anorexia, and dehydration	12	29.3
High fever, lethargy, and yellow to greenish-brown diarrhea	07	17.1
Mild fever and blood-streaked diarrhea	04	9.7
Mild fever, lethargy, gaseous stomach, and diarrhea	03	7.3

Significant differences in the prevalence of *Salmonella* spp. among different breeds of goats were determined (Table-13). Crossbreeds (57.1%; 95%CI: 40.9-72.3) were found to be significantly high prevalent to *Salmonella* spp. than Black Bengal (44%; 95%CI: 24.4-65.1) and Jamunapari (4.5%; 95%CI: 1.7-9.6) breeds of goat. The prevalence of *Salmonella* spp. was significantly higher (p≤0.01) in goats with good BCS (28.6%; 95%CI: 18.8-40%) than fair (20.3%, 95 CI: 11.6-31.7) and poor (9.3%, 95% CI: 3.1-20.3) BCS. The goats from the family sources (23.9%, 95% CI: 17.3-31.7) were more prevalent than that of goats from a farm (11.1%, 95% CI: 4.2-22.6). In the case of age, adult goats (27.8%; 95% CI: 14.2-45.2) were more prevalent than young (18.3%, 95% CI: 10.1-29.3) and juvenile (19.4%, 95% CI: 11.9-28.8). Although it was not statistically significant, male (23.5%, 95% CI: 14.7-34.2) showed highly prevalent than the female (18.5%, 95% CI: 11.9-26.6).

**Table-13:** Factors influencing the infection of *Salmonella* spp. in goats.

Variables	Categories	*Salmonella* spp.			Multiple logistic regression		
	
		n (%)	95% CI	p (c^2^-test)	OR	95% CI	p-value
Season	Winter (120)	21 (17.5%)	11.2-25.5	0.19	1		
	Summer (80)	20 (25%)	15.9-35.9		1.8	0.7-4.4	0.24
Breed	Jamunapari (133)	6 (4.5%)	1.7-9.6	**<0.01**	1		
	Black Bengal (25)	11 (44%)	24.4-65.1		22.8	6.5-79.9	**<0.01**
	Cross (42)	24 (57.1%)	40.9-72.3		27.3	9.1-81.8	**<0.01**
Sex	Female (119)	22 (18.5%)	11.9-26.6	0.39			
	Male (81)	19 (23.5%)	14.7-34.2				
Source	Farm (54)	6 (11.1%)	4.2-22.6	**0.04**	1		
	Family (146)	35 (23.9%)	17.3-31.7		3.1	0.9-9.5	0.05
Age	Juvenile (0 days-1 year) (93)	18 (19.4%)	11.9-28.8	0.48			
	Young (1 year-2 years) (71)	13 (18.3%)	10.1-29.3				
	Adult (>2 years) (36)	10 (27.8%)	14.2-45.2				
Body condition score	Poor-1 (54)	5 (9.3%)	3.1-20.3	0.02	1		
	Fair-2 (69)	14 (20.3%)	11.6-31.7		2.2	0.5-8.4	0.26
	Good-3 (77)	22 (28.6%)	18.8-40		5.7	1.5-21.5	**0.01**
Vaccination	Yes (14)	7 (50%)	23.1-76.9	**<0.01**	6	1.2-31.3	**0.03**
	No (186)	34 (18.3%)	13-24.6		1		

In multivariable logistic regression analysis of significant factors, it was found that the prevalence of *Salmonella* spp. was 27.3 times higher in crossbreeds and 22.8 times higher in Black Bengal goats than the Jamunapari goats breed. The odds of prevalence of *Salmonella* spp. was significantly higher in goats with good BCS (OR=5.7, CI: 1.5-21.5, *P*=0.01) than the fair and poor BCS goats. The prevalence of *Salmonella* spp. was 3.1 times higher in goats reared in a family than the goats reared in farms (Table-13).

### AMR pattern of *Salmonella* spp. in goats

**Figure-5:**
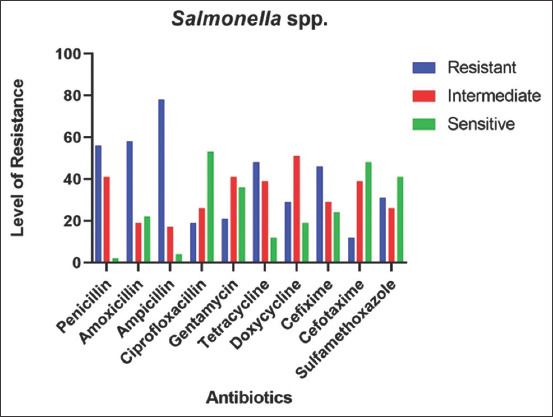
Antimicrobial resistance pattern of *Salmonella* spp.

In AMR and sensitivity testing, *Salmonella* spp. was found to be highly resistant against Ampicillin (78%), Amoxicillin (59%), and Penicillin (56%) whereas, Cefotaxime (12%) was the lowest resistance to among all drugs tested. Ciprofloxacin showed the highly sensitive (54%) and Penicillin (2%) showed the least sensitive among all drugs. Doxycycline (51%) showed moderate resistance against *Salmonella* spp., followed by Gentamicin (42%) and Penicillin (42%) against *Salmonella* spp. isolated from goats (Figure-5).

## Discussion

In developing countries like Bangladesh, livestock plays an essential role in food security, poverty alleviation, and indeed a token of people’s livelihoods [19]. However, infectious diseases of animals are causing major economic losses of farmers inflicted by hampering production. Moreover, many of them are zoonotic in nature which increases the significance of it both animal and human health. Although vaccination and good hygienic practices are among the most effective measures to prevent these diseases, antibiotics are extensively used either as prophylactic agents, or therapeutics in the livestock industry in Bangladesh. The widespread use of antimicrobial agents contributes to the development of AMR too. Therefore, the importance of rapid confirmatory diagnosis and AMR pattern of pathogens became a key concern for the effective treatment, as well as the prevention and control of these infectious diseases.

In the present study, confirmatory diagnosis of some infectious diseases having non-specific clinical signs such as diarrhea and/or fever was done using a rapid molecular diagnosis kit to establish their diagnostic and treatment protocol in goats in SAQTVH, CVASU. They were *S. aureus, C. perfringens, L. monocytogenes, and Salmonella* spp. Overall proportionate prevalence of *S. aureus* infection in goats of Chattogram Metropolitan area was found to be 11.5%. A similar study was conducted by Zaman *et al*. in SAQTVH and reported a 14% prevalence of *S. aureus* in goats [20]. Another group in other parts of Bangladesh reported somewhat higher prevalence (26%) of *S. aureus* [21]. The researcher reported variable results in other parts of the world such as 43.24% in China [22], 44% in Egypt [23], 39.7% in Nigeria [24], and 30% in West African dwarf goats [25]. This variation may be due to different geographical locations and environmental conditions.

The present study determined sex as a potential risk factor for *S. aureus* infection in goats (OR= 2.6, female vs. male) which is coincide with the findings in Bangladesh [20]. However, these findings differ from Nigeria where they reported more prevalence of *S. aureus* infection in males compared to the females [24]. This current study also identified age as a significant risk factor (OR=6, adult vs. young and juveniles) which is also similar to the previous findings [20].

Different types of clinical signs were noticed in this study in the case of *S. aureus* infection in goats. Common clinical signs encountered such as mild fever, nasal discharge, dyspnea, and coughing whose are obvious as it causes respiratory infection. These clinical signs also match with findings reported in an earlier report [21]. *S. aureus* is also responsible for the sub-clinical mastitis in goats where the clinical signs are limited [26]. This study also reported some clinical signs such as fever, lethargy, dehydration, and loss of appetite in recently parturated goats that may be due to sub-clinical form of mastitis.

AST showed that most of the *S. aureus* ­isolates were resistant against the majority of antibiotics and the sensitivity rates below 40%, with exceptions of Amoxicillin (48%), Ciprofloxacin (44%), and Gentamicin (44%). *S. aureus* showed relatively high resistance to Penicillin, Ampicillin, Tetracycline, Doxycycline, Cefixime, and Cefotaxime which is near to the resistance pattern of *S. aureus* isolated from sheep and goats in China and Spain [22,27]. The present study revealed Amoxicillin, Ciprofloxacin, and Gentamicin as relatively sensitive and Cefotaxime, Tetracycline, and Cefixime as resistant against *S. aureus* which is similar to the findings reported previously [28].

*C. perfringens* toxinotypes are responsible for enterotoxemia in goat. In this study, goat patients at SAQTVH from different regions of Chattogram Metropolitan area were screened for the presence of *C. perfringens* type D. Our findings revealed that 7 (3.5%) out of 200 goats were positive for *C. perfringens* by PCR amplification. In accordance with our study, a higher prevalence of *C. perfringens* in goats of India (60%) and Pakistan (66.5%) has been recorded [29]. In this study, we spot sex as a key risk factor for *C. perfringens* in goats (OR=5.6 male vs. female) which contradicts with previous findings where they found more prevalence (15%) in female than male goats [30]. In case of age, this study recognizes juvenile and young goats are more susceptible to *C. perfringens* infection than adult goats, which is supported by the findings previously reported [30,31]. The most frequent clinical signs of *C. perfringens* infected goats were diarrhea, convulsion, abdominal pain, and incoordination which are supported by previous findings where they observed neurological signs along with abdominal discomfort and diarrhea [30,32].

The beta-lactams are commonly used for the treatment of *C. perfringens* associated diseases. In our study, we also found that *C. perfringens* is susceptible to Penicillin (57%), which is consistent with some previous findings [33]. Gentamicin (71%) and Ciprofloxacin (57%) were found to be highly resistant against *C. perfringens*, which is similar to the finding described earlier [34].

The prevalence of listeriosis has not been well reported in goats of Bangladesh. Therefore, we aimed to determine the prevalence of *L. monocytogenes* in goats. The overall prevalence of *L. monocytogenes* in the present study was 1% which is similar to some of the studies conducted at home and abroad where they recorded that the prevalence of *L. monocytogenes* in small ruminants is lower in comparison to other infectious causes [35-37]. However, study reported the higher prevalence of *L. monocytogenes* (16.66%) in India, which may be due to inadequate hygienic conditions and low ambient temperature during the period of sampling and processing [38].

Due to the low prevalence, it was difficult to identify the risk factors for *L. monocytogenes* in goats. In the present study, both the positive cases were found in winter and in goats that were reared in farms where silage was supplied. This may be due to the organism is more prevalent in winter and transmitted through silage [39]. Clinical manifestations of invasive listeriosis in ruminants are usually severe. In this study, we found protrusion of tongue and incoordination in listeria- affected goats supporting the findings of encephalitic listeriosis in small ruminants [40]. However, this is difficult to conclude with this sample size and identified cases. Further study may need to know the detail on *L. monocytogenes*.

Antibiogram study of *L. monocytogenes* isolates exhibited high sensitivity against Penicillin and Cefixime and resistance against Amoxicillin and Ampicillin, which contradict with one study [41] but is consistent with another [42]. The present study showed that Ciprofloxacin, Doxycycline, and Tetracycline are intermediately sensitive, whereas [38] spot 100% sensitivity of Ciprofloxacin against *L. monocytogenes*.

The overall prevalence rate of *Salmonella* spp. in goats in Chattogram Metropolitan area was recorded as 20.5%, which is significant and should not be overlooked as its public health significance and the high possibility of dissemination of diseases in man, animals, and birds. The prevalence rate of *Salmonella* spp in goats of this study is near to the findings of some other studies conducted in Bangladesh as well as abroad [20,43,44]. On the other hand, a very low prevalence (0.1%) was reported in adult diarrheic goats [45].

This study recognized the breed as a potential risk factor for the infection of salmonella in goat (OR=27.3, cross vs. Jamunapari) and (OR=22.8, Black Bengal vs. Jamunapari). The high prevalence of *Salmonella* spp. in Black Bengal and Crossbreeds in comparison to Jamunapari was also identified previously [20]. In this study, the prevalence of *Salmonella* spp. was somewhat higher in adult goats compare to the young, which is supported by one study [20] but in contrast to the findings of another study [44]. This study also identified the source of animals as a potential risk factor (OR=3.1, family vs. farm), family livestock is much more susceptible to salmonellosis due to poor hygienic management in comparison to farm, which is similar to the findings previously reported [46].

Salmonellosis is one of the important diseases that cause diarrhea in goats. Three common conditions caused by *Salmonella* are gastroenteritis, enteric fever, and bacteremia. In this study, fever, anorexia, dehydration, and mucus in feces were the most commonly encountered in goats infected with salmonella, but few other signs were also observed. Clinical signs recorded in this study are in accordance with signs mentioned by Radostits *et al*. [31].

All the Salmonella isolates were tested against ten antibiotics of different groups. The highest sensitivity of Ciprofloxacin indicates that fluoroquinolones still be the first choice of salmonella infected patient. Chloramphenicol was also suggested as a drug of choice in salmonellosis in goats [46]. In this study, Salmonellae isolates were highly sensitive to Ciprofloxacin, Cefotaxime, and Sulfamethoxazole, intermediately sensitive against Gentamicin and Doxycycline, and resistant against Penicillin, Amoxicillin, and Tetracycline, which is close to the several findings reported in home and abroad [43,47].

## Conclusion

Infectious diseases are an important constraint for the development of goat farming in Bangladesh. In this study, the prevalence of *S. aureus*, *C. perfringens, L. monocytogenes*, and *Salmonella* spp. was identified as 11.5%, 3.5%, 1%, and 20.5%, respectively in goats. Females were found to be more susceptible to *S. aureus* infection in goats than males. Male goats and Crossbreeds acted as potential risk factors and significantly higher contributor to the occurrence of *C. perfringens* infection in goats. Black Bengal and Crossbreeds along with family goats had significantly higher contribution to the occurrence of salmonellosis in goats. Ciprofloxacin found to be sensitive against *S. aureus* (44%) and *Salmonella* spp. (54%) but resistant against *C. perfringens* (57%). Penicillin showed sensitivity against *C. perfringens* (57%) and *L. monocytogenes* (100%) infection in goats whereas resistance against *S. aureus* (62%) and *Salmonella* spp. (56%). Amoxicillin was highly sensitive against *S. aureus* (48%) and resistant to *Salmonella* spp. (59%). Rapid diagnostic methods such as multiplex PCR were found to be effective for the confirmation of specific pathogens from goats showing non-specific clinical signs. This present study is also able to identify some potential risk factors responsible for those diseases and antimicrobials that were effective against those organisms.

## Recommendations

Due to time and resource limitations, we conducted the study on a small scale. In the future, the study can be conducted involving a higher sample size. The current study proposes some common signs for the diagnosis of staphylococcosis, clostridiosis, listeriosis, and salmonellosis in goats which is based on the molecular detection of organisms.

## Authors’ Contributions

PP, MRF, and MYEC designed the study, wrote the manuscript, and participated in conducting the experiment. PP and PD collected the samples. PP performed the laboratorial investigations. PP, MYEC, and MKR processed and analyzed the data. All authors read and approved the final manuscript.
